# A pyrosequencing-based metagenomic study of methane-producing microbial community in solid-state biogas reactor

**DOI:** 10.1186/1754-6834-6-3

**Published:** 2013-01-15

**Authors:** An Li, Ya’nan Chu, Xumin Wang, Lufeng Ren, Jun Yu, Xiaoling Liu, Jianbin Yan, Lei Zhang, Shuangxiu Wu, Shizhong Li

**Affiliations:** 1 Institute of Nuclear and New Energy Technology, Tsinghua University, Tsinghua Garden, Haidian District, 100084, Beijing, China; 2 The CAS Key Laboratory of Genome Sciences and Information, Beijing Institute of Genomics, Chinese Academy of Sciences, No.1-7 Beichen West Road, Chaoyang District, 100101, Beijing, China

**Keywords:** Solid-state fermentation, Biogas production, Pyrosequencing, Metagenomics, DNA extraction, *Anaerococcus*, *Psychrobacter*

## Abstract

**Background:**

A solid-state anaerobic digestion method is used to produce biogas from various solid wastes in China but the efficiency of methane production requires constant improvement. The diversity and abundance of relevant microorganisms play important roles in methanogenesis of biomass. The next-generation high-throughput pyrosequencing platform (Roche/454 GS FLX Titanium) provides a powerful tool for the discovery of novel microbes within the biogas-generating microbial communities.

**Results:**

To improve the power of our metagenomic analysis, we first evaluated five different protocols for extracting total DNA from biogas-producing mesophilic solid-state fermentation materials and then chose two high-quality protocols for a full-scale analysis. The characterization of both sequencing reads and assembled contigs revealed that the most prevalent microbes of the fermentation materials are derived from Clostridiales (Firmicutes), which contribute to degrading both protein and cellulose. Other important bacterial species for decomposing fat and carbohydrate are Bacilli, Gammaproteobacteria, and Bacteroidetes (belonging to Firmicutes, Proteobacteria, and Bacteroidetes, respectively). The dominant bacterial species are from six genera: *Clostridium*, *Aminobacterium*, *Psychrobacter*, *Anaerococcus*, *Syntrophomonas*, and *Bacteroides*. Among them, abundant *Psychrobacter* species, which produce low temperature-adaptive lipases, and *Anaerococcus* species, which have weak fermentation capabilities, were identified for the first time in biogas fermentation. Archaea, represented by genera *Methanosarcina*, *Methanosaeta* and *Methanoculleus* of Euryarchaeota, constitute only a small fraction of the entire microbial community. The most abundant archaeal species include *Methanosarcina barkeri fusaro*, *Methanoculleus marisnigri JR1*, and *Methanosaeta theromphila*, and all are involved in both acetotrophic and hydrogenotrophic methanogenesis.

**Conclusions:**

The identification of new bacterial genera and species involved in biogas production provides insights into novel designs of solid-state fermentation under mesophilic or low-temperature conditions.

## Background

Due to fossil fuel crisis, atmospheric pollution, and global warming, the development of renewable and clean energy forms has become a critical task for the human society. The production of biogas through biomass fermentation, regarded as an environment-friendly, clean, and renewable resource, has been gaining more attention in many developed and developing countries [1,2]. In China, solid biomass wastes (SW), such as kitchen, livestock, and agricultural wastes (largely crop straws and stalks), are produced at the multi-million ton level annually [3] and the untreated disposals of such wastes may lead to severe long-term environmental hazards and resource wasting. Therefore, the utilization of anaerobic fermentation to convert SW into biogas represents a promising effort if it can be accomplished at an industrial scale and in an economical way. In recent years, solid-state anaerobic digestion (SS-AD) has been promoted in China because of its many advantages, including less reactor-capacity demand, lower heating-energy need, and no stirring-energy consumption, particularly as opposed to liquid-state anaerobic digestion [2]. However, the yield of methane, the major end-product of this process, has not been sufficient for an industrial-scale promotion, let alone economical plausibility.

The biochemical process for anaerobic methane production is complex. The diversity and abundance of microbes involved in the process certainly play a major role, which are influenced by microbial community compositions, fermentation materials, climate variations, and designs of chambers, to name just a few. In the initial steps of SS-AD, hydrolytic Firmicutes reduce large macromolecules (including proteins, complex fats, and polycarbohydrates) to their building blocks (i.e., amino acids, long-chain fatty acids, and monosugars) and other bacteria (including acidogens and acetogens) further degrade them into smaller intermediates (such as acetate, carbon dioxide, and hydrogen). In the later steps, methanogens, which are mainly derived from Archaea, convert the smaller substrates into methane through both aceticlastic and hydrogenotrophic pathways [4-6]. Therefore, a thorough understanding of composition, structure, and function of the microbial communities residing in anaerobic reactors is crucial for developing novel fermentation strategies and improving methane yield of the existing biogas reactors as well as ideas for novel designs.

Although the biochemistry and enzymology of methanogenesis for model organisms are well characterized [7], the structure and function of biogas-producing microbial communities have not been sufficiently explored, particularly under different anaerobic fermentation conditions. In the past decade or so, investigations of different biogas-producing systems and waste treatment conditions, including anaerobic mesophilic sludge digester [8], mesophilic anaerobic chemostats fed with synthetic wastewaters [9,10], thermophilic upflow anaerobic filter reactor [11], fully- stirred reactor fed with fodder beet silage [12], thermophilic municipal biogas plant [13], and two-phase liquid biogas reactor operated with silages [14], have been conducted. Thermophilic anaerobic municipal solid-waste digester [15] and packed-bed reactor for degrading organic solid wastes of artificial garbage slurry [16] were also studied. However, the methodology used for these studies was based on constructing and sequencing 16S rDNA and *mcrA* clone libraries, and the choice of PCR primers for amplifying sequence fragments of the target genes and other sequences typically creates biases, and it has been difficult to cover the entire complexity of microbial communities based on just the sequences from a limited number of gene-specific clones. The next-generation sequencing technologies have overcome many of these problems, particularly the pyrosequencing platform (such as the Roche/454 GS FLX sequencer) that generates longer read lengths ranging from 200 to 400 bp as compared to other platforms (such as the Illumina Hiseq2000 system that generates 50–150 bp reads in its single-directional sequencing runs [17-20]) and creates less bias in sequencing library construction [21,22]. Based on this platform, a German group conducted the first metagenomic analysis on a complex system of biogas-producing plant [20], and developed related bioinformatic methods [23,24]. They further revealed that in addition to the archaeal methanogen *Methanoculleus* species (which play a dominant role in methane production) and abundant numbers of cellulolytic *Clostridia* (which were important for the hydrolysis of cellulosic plant biomass for acetogenesis) other methanogen taxa (including *Streptococcus*, *Acetivibrio*, *Garciella*, *Tissierella*, and *Gelria*) are also detected but their precise functional roles in methane formation remain to be elucidated [17,25]. A similar study that used a SOLiD™ short-read DNA sequencing platform has recently confirmed the importance of hydrogen metabolism in biogas production [26]. Nevertheless, a metagenomic study on the SS-AD system based on deep-sampling and long-read sequencing supported by the next-generation sequencing platforms is of essence in moving the field forward.

Aside from sequencing and bioinformatic analysis, DNA extraction and its quality yield from samples of complex materials (such as liquid vs. solid and source vs. processing) also greatly affect results of metagenomic sequencing [27,28]. DNA extraction efficiency and quality from biogas samples have also been compared to PCR-based analyses [29], but a robust method, particularly for analyzing samples from SW biogas fermentation materials and based on high-throughput shotgun pyrosequencing, has yet to be reported. Toward this end, we first evaluated five DNA extraction protocols (including four based on commercial kits and one derived from the classic chloroform-isoamylalcohol method) for samples collected from a mesophilic SS-AD fermenter fed with SW. After the T-RFLP evaluation (see Methods for details), we then chose the two better protocols and prepared DNA samples for our pyrosequencing-based metagenomic study. Our results have led to novel insights into microorganism composition, gene content, and metabolic capacity of the SW fermentation.

## Results and discussion

### Evaluation of DNA extraction methods for high-throughput pyrosequencing

**Figure 1:**
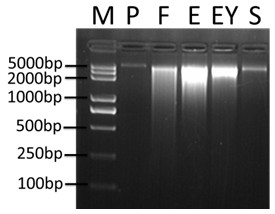
**The electrophoresis results of DNA preparations based on the five methods from a biogas reactor sample.** M, molecular marker Transplus 2 K. P, F, E, EY, S refer to the five protocols, respectively. Except for Protocol P, which was loaded with 5 μl of undiluted DNA solution, only 1 μl of undiluted DNA solution was loaded on the 0.8% agarose gels for the rest of the extracts.

Biogas fermentation samples are extremely complex due to the presence of multiple organic compounds and diverse degradation products. In SS-AD samples, microorganisms bind strongly to solid materials and have a rather heterogeneous distribution inside the samples. In order to find a better protocol for the isolation of high-quality DNA preparations for pyrosequencing, we set out to evaluate five DNA extraction methods. Using electrophoresis assay for checking quality and yield of genomic DNA extracts (Figure 1 and Table 1), we found that Protocols E, EY, and F gave rise to the highest yields, ranging from ~160.5 ng/μl to ~121.4 ng/μl, while Protocol P produced the lowest yield, with ~20.5 ng/μl. However, Protocol F showed the highest degree of smearing (Figure 1) and both Protocols F and S showed low purity based on A_260_/A_230_ ratios (Table 1). The DNA extract from Protocol S appeared dark yellowish and its quality could not be measured based on spectrophotometry. The differences among the five methods were primarily observed at the cell lysing steps, which are critical for DNA yield and quality especially when field sampling is the only source [29,30]. According to our results, Protocol P (the Mo-Bio PowerSoil DNA Isolation Kit) showed the lowest DNA yield, suggesting insufficient lysing despite the use of vigorous mechanical force (vortexing for 15 min), especially when compared with the corresponding steps in other related protocols (hand shaking for a few minutes or vortexing for 30 s). Therefore, we realized that in addition to mechanical forces, lysis reagents used for the protocols may also be crucial for preparing better cell lysis.

**Table 1:** Comparison of DNA yield and purity among five DNA extraction protocols

**Method**	**Parameters of DNA quantity and purify **^**$**^		
	**DNA yield (ng/μl)**	**A**_**260**_**/A**_**280**_	**A**_**260**_**/A**_**230**_
P	20.5	1.61	0.88
F	153.6	1.81	0.33
E	160.5	1.88	1.89
EY	121.4	1.81	1.82
S	ND	ND	ND

^$^ DNA quantity and purity were measured fluorometrically based on NanoDrop. The purity parameter concerning carbohydrate, phenol and aromatic compound contaminations was calculated based on the ratio of the absorptions at 260 nm to 230 nm (A_260_/A_230_). To check for protein contamination, the ratio of the absorptions at 260 nm to 280 nm was evaluated (A_260_/A_280_). ND, not determined due to the impurity of DNA solution. The purity of DNA preparations was measured based on A_260_/A_280_ > 1.8 and A_260_/A_230_ > 1.8.

We further evaluated the DNA preparations from all five methods based on T-RFLP analysis. The Shannon-Weiner index was used to indicate diversity and complexity, and the Simpson index was used to measure abundance. Bacteria and archaea were analyzed separately. The results showed that Protocol E resulted in the highest bacterial diversity (Shannon-Weiner index of 3.6) and the highest abundance (Simpson index of 0.95; Table 2), followed by Protocols P and EY. Protocols E and EY showed higher archaeal enrichment than that of Protocols P, F and S. We therefore chose DNA extracts from Protocols E and EY for pyrosequencing, which consistently lead to higher yield, purer DNA, and high microbial diversity.

**Table 2:** Diversity indexes of T-RFLP analysis for different protocols

**Method**	**Diversity index for bacteria**		**Diversity index for archaea**	
	**Shannon- Weiner Index (H)**	**Simpson Index (D)**	**Shannon- Weiner Index (H)**	**Simpson Index (D)**
E	3.578163	0.945856	2.76696	0.88734
F	3.293445	0.923434	1.245221	0.561454
P	3.410967	0.944772	1.984324	0.762865
S	3.116197	0.921699	1.634894	0.665427
EY	3.313463	0.924693	2.670502	0.872466

For each protocol, bacterial and archaeal diversities were analyzed separately.

### Sequencing and metagenomic assembly

**Figure 2:**
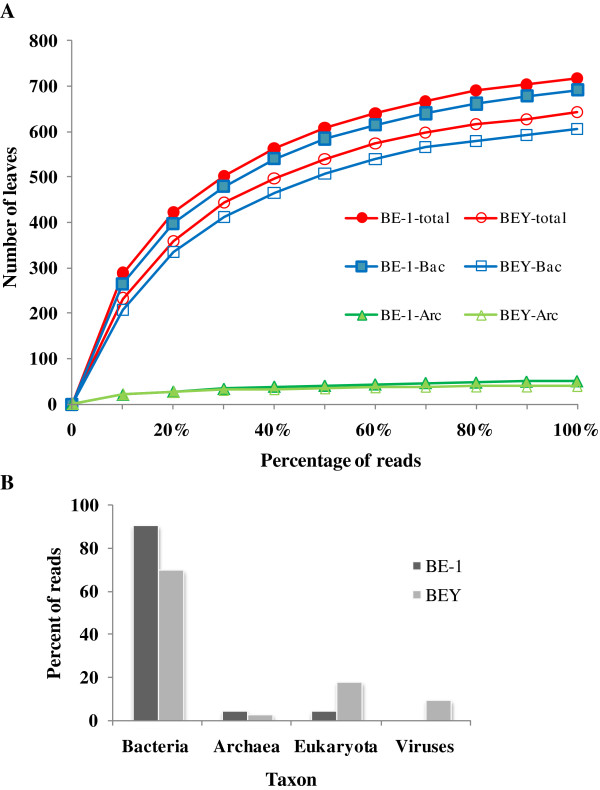
**Rarefaction curves (A) and the relative proportions of taxonomic classification of bacteria, archaea, eukaryota, and viruses (B) from the BE-1 and BEY libraries.** For the rarefaction curves, the analysis was performed on the total (including bacteria, archaea, eukaryota, viruses and environmental sequences), archaeal, and bacterial taxa from the two libraries.

Pyrosequencing of two DNA libraries (from Protocols E and EY), namely “BE” and “BEY”, were performed and the data from the experiments were summarized in Table 3. The first sequencing runs of BE (named as BE-1) and BEY resulted in 266,781,751 bp sequences from 738,005 reads (an average read length of 362 bp) and 197,514,392 bp sequences from 551,339 reads (an average read length of 358 bp), respectively. It is obvious that there are more data and higher microbial richness (Figure 2A in section 3.3) obtained from BE (BE-1) than from BEY. Therefore, the BE sample was sequenced twice again as BE-2 and BE-3.

**Table 3:** Summary of sequencing results

	**BEY**	**BE-1**	**BE-2**	**BE-3**	**BE (total)**
**Number of reads**	**551,339**	**738,005**	**781,293**	**761,304**	**2,280,601**
**Number of bases**	**197,514,392**	**266,781,751**	**218,761,141**	**161,838,822**	**647,369,218**
**Average read length (bp)**	**358**	**362**	**280**	**212.58**	**283**
**Number of large contig (>500nt)**	**11897**	**17,933**	**10,861**	**7,354**	**37,276**
**Number of all contig**	**24,052**	**33,750**	**20,281**	**17,860**	**118,433**
**Max contig length**	**18,435**	**35,598**	**35,601**	**11,781**	**158,075**
**Number of bases in all contigs**	**16,193,286**	**25,185,216**	**16,441,312**	**11,362,543**	**76,759,543**
**Assembled bases %**	**8.2%**	**9.44%**	**7.5%**	**7%**	**11.9%**
**N50 (large contigs) (bp)**	**1105**	**1204**	**1440**	**1231**	**1712**

The BE and BEY sequences were assembled separately by using GS de novo assembler.

Since the BE sample was sequenced three times, it yielded 647,369,218 bp sequences from 2,280,601 reads (in an average read length of 283 bp). The assembly of the total reads gave rise to 118,433 contigs containing 76,759,543 bp, which were accounted for approximately 12% of the total sequences measured in basepairs generated in this study. The number of large contigs (>500 nt) was 37,276 (an N50 of 1,712 bp), in which the largest contig contains 158,075 bp. The average GC content of the total reads from the BE sample is 46% (Additional file 1: Figure S1).

### Comparison of microbial compositions between samples BE-1 and BEY

We used rarefaction analysis to assess species richness of the system. Using MEGAN (a meta-genomic bioinformatic tool) and at the best resolved levels based on the NCBI taxonomy database and our sequence data, we analyzed the microbial richness, based on sequence reads, between libraries BE-1 and BEY (Figure 2A) and revealed that the number of taxonomic leaves or clades of BE-1 are all higher than those of BEY, and the result indicated that BE-1 contains more microbial taxa than BEY, and indeed BE-1 and BEY contain 717 and 643 leaves for all assigned taxa, respectively. Furthermore, the rarefaction curves of both libraries in archaea appear close to saturation at 20% of the total reads, whereas those in bacteria are increased to 100% of the total reads. Our results suggest that the current sampling depth is not yet close to the natural status for bacteria but may be saturated for archaea.

Matching the sequencing reads from BE-1 and BEY to sequences collected in NT and NR databases, we dissected microbial community structure of the two libraries (excluding the reads with no-hits; Figure 2B), showing that at the domain level there is significant difference between the two libraries in the proportion of reads assigned to bacterial, archaeal, viral, and eukaryotic sequences (Figure 2B). In the BE-1 data set, 4.7% and 90.9% of the reads were assigned to archaea and bacteria, but decreased to 3.0% and 71.2% for those of BEY, respectively. In contrast, only 3.4% of the reads were assigned to eukaryotes and almost no viral sequence was detectable in BE-1, but eukaryotic and viral detections were significantly increased to 20.5% and 9.3% in BEY, respectively. The taxonomic bias in the microbial communities detected between the datasets from Protocols E and EY may reflect the thoroughness of sample pre-washing with TENP buffer that may partially wash off bacteria known to be lightly adhered to solid matrix.

Examining the taxonomies built from mapped single reads of libraries BE-1 and BEY (Additional file 1: Figure S2), we observed that the dominant taxa at the genus level for archaea and bacteria (such as *Methanosarcina* and *Clostridium* for the former and the latter, respectively) are comparable between the two libraries; but there were greater numbers of microbial taxa observed in the BE (combining BE-1, BY-2, and BE-3) and BEY libraries (Table 2 and Figure 2A). Therefore, Protocol E in combination with the E.Z.N.A.TM Soil DNA Kit should be considered as the most suitable procedure for the SW fermentation samples.

### Microbial composition analysis based on sequencing reads and assembled contigs

**Figure 3:**
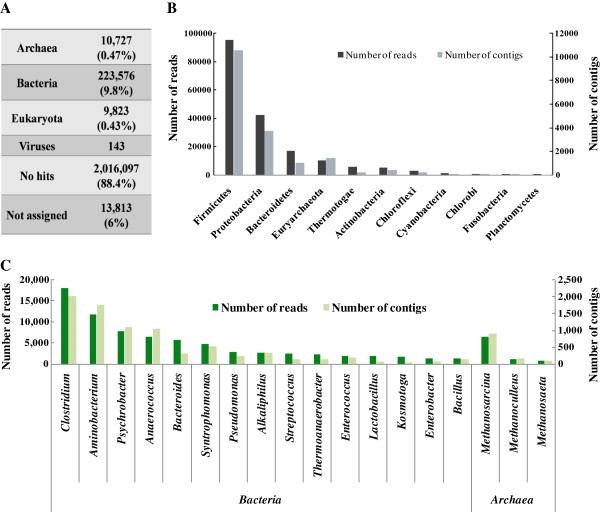
**The statistics for the reads assigned (A), the number of reads/contigs for the 11 most prevalent phyla (B), and the number of reads/contigs of 15 most prevalent genera in bacteria and the 3 most prevalent genera in archaea (C) obtained in the taxonomic classification analysis using BLASTN/BLASTX tools against the GenBank NT/NR database with a E-value cutoff of 10**^**-5 **^**based on total reads/contigs.**

We analyzed the microbial community composition of the BE library using MEGAN and mapped individual reads first. Figure 3 shows the statistics of the total assigned reads and their annotations for the popular genera and phyla. The reads assigned to the superkingdoms Bacteria (~9.8%) and Archaea (~0.5%) were accounted for approximately 10.3% of the total reads, whereas 88.4% of the total reads obtained have no hit in the present database, indicating that there are still an immense amount of unknown/uncultured species in this complex anaerobic biogas-producing sample (Figure 3A).

Based on single-read assignments (Figure 3B), the most prevalent bacterial taxa at the phylum level are Firmicutes (39.0% of hit-reads), followed by Proteobacteria (17.3% of hit-reads) and Bacteroidetes (7.0% of hit-reads), which are responsible for biomass degradation and fermentation. The 4^th^ most abundant taxon is Euryarchaeota (4.3% of hit-reads), involved in methane synthesis and taking a small fraction of the community. In addition, massive bacterial taxa are distributed in phyla Thermotogae (2.4% of hit-reads), Actinobacteria (2.2% of hit reads), Chloroflexi (1.2% of hit-reads), Cyanobacteria (0.4% of hit-reads), Chlorobi (0.3% of hit-reads), and Fusobacteria (0.3% of hit-reads), and the result again indicates that there are more complex microbial components residing within this system and that some of the components may reflect their original environments rather than characteristic of the SW feeds in general.

At the class level, the prevalent reads are distributed over Clostridia (66,455 reads, 27.2% of hit-reads), Bacilli (16,767reads, 6.9% of hit-reads), Gammaproteobacteria (19,085 reads, 7.8% of hit-reads), Bacteroidetes (11,765 reads, 4.8% of hit-reads), and Methanomicrobia (9180 reads, 3.8% of hit-reads), which belong to phyla Firmicutes, Proteobacteria, Bacteroidetes, and Euryarchaeota, respectively (Table 4).

**Table 4:** The 21 most prevalent genera of taxonomic classification of sample BE based on read counts

**Phylum**	**Class**	**Order**	**Family**	**Genus**	**Counts of reads**
Firmicutes	Clostridia	Clostridiales	Clostridiaceae	***Clostridium***	17,975
	Clostridia	Clostridiales	Syntrophomonadaceae	***Aminobacterium***	11,870
	Clostridia	Clostridiales	Peptostreptococcaceae	***Anaerococcus***	6,544
	Clostridia	Clostridiales	Syntrophomonadaceae	***Syntrophomonas***	4,698
	Clostridia	Clostridiales	Clostridiaceae	***Alkaliphilus***	2,637
	Bacilli	Lactobacillales	Enterococcaceae	***Enterococcus***	1,948
	Bacilli	Lactobacillales	Streptococcaceae	***Streptococcus***	2,462
	Clostridia	Clostridiales	Syntrophomonadaceae	***Thermanaerovibrio***	1,123
	Bacilli	Bacillales	Bacillaceae	***Bacillus***	1,282
	Clostridia	Thermoanaerobacteriales	Thermoanaerobacteriaceae	***Thermoanaerobacter***	2,302
	Bacilli	Lactobacillales	Lactobacillaceae	***Lactobacillus***	1,907
Bacteroidetes	Bacteroidetes	Bacteroidales	Bacteroidaceae	***Bacteroides***	5,655
	Bacteroidetes	Bacteroidales	Porphyromonadaceae	***Parabacteroides***	1,295
Proteobacteria	Gammaproteobacteria	Pseudomonadales	Moraxellaceae	***Psychrobacter***	7,823
	Gammaproteobacteria	Pseudomonadales	Pseudomonadaceae	***Pseudomonas***	2,850
	Gammaproteobacteria	Enterobacteriales	Enterobacteriaceae	***Enterobacter***	1,339
Chloroflexi	Chloroflexi	Chloroflexales	Chloroflexaceae	***Roseiflexus***	968
Thermotogae	Thermotogae	Thermotogales	Thermotogaceae	***Kosmotoga***	1,738
Euryarchaeota	Methanomicrobia	Methanosarcinales	Methanosarcinaceae	***Methanosarcina***	6,522
	Methanomicrobia	Methanomirobiales	Methanomicrobiaceae	***Methanoculleus***	1,102
	Methanomicrobia	Methanosarcinales	Methanosaetaceae	***Methanosaeta***	750

# Read counts matched to the 21 most abundant microbial genera. The results were obtained from the best BLASTx hits by searching the nucleotide sequence database of NCBI GenBank.

At the genus level, there are 429 genera of bacterial and 39 genera of archaeal origins. The six most prevalent genera for bacteria are *Clostridium* (17,975 reads, 7.4% of hit-reads), *Aminobacterium* (11,870 reads, 5.2% of hit-reads), *Psychrobacter* (7,823 reads, 4.9% of hit-reads), *Anaerococcus* (6,544 reads, 2.7% of hit-reads), *Bacteroides* (5,655 reads, 2.3% of hit-reads), and *Syntrophomonas* (4698 reads, 1.9% of hit-reads). For archaeal species, the three most prevalent genera are *Methanosarcina* (6,522 reads, 2.7%), *Methanoculleus* (1,102 reads, 0.5%), and *Methanosaeta* (750 reads, 0.3%), which all belong to class Methanomicrobia of Euryarchaeota (Table 4, Figure 3C).

**Figure 4:**
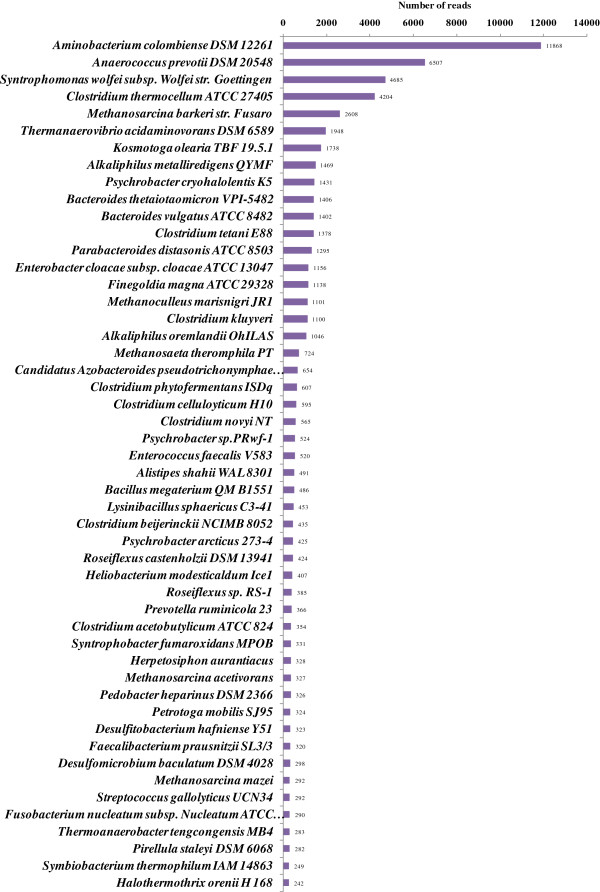
**Statistics for the reads assigned to microbial genome sequences using BLASTN/BLASTX tools against the GenBank NT/NR database with an E-value cutoff of 10**^**-5 **^**based on the total reads.** The x-axis denotes the number of reads assigned to the 50 most prevalent microbial strain genomes.

At the species level (Figure 4), although *Clostridium* is the predominant genus, the three most abundant bacterial species are *Aminobacterium colombiense DSM 12261* (11,868 reads, 5.2%), *Anaerococcus prevotii DSM 20548* (6,507 reads, 2.9%), *Syntrophomonas wolfei subsp*. *Wolfei str*. *Goettingen* (4,685 reads, 1.9%), while the dominant *Clostridium* species were identified as *C*. *thermocellum* ATCC 27405 (4,204 reads, 1.7%), *C*. *tetani* E88 (1,378 reads, 0.6%), *C*. *kluyveri* (1,100 reads. 0.5%), and *C*. *phytofermentans* ISDg (607 reads, 0.3%). In archaea, the three most prevalent species are *Methanosarcina barkeri str*. *Fusaro* (2,611 reads, 1.1%), *Methanoculleus marisnigri JR1* (1,101 reads, 0.5%), *Methanosaeta theromphila* PT (724 reads, 0.3%).

Meanwhile, we also analyzed microbial community compositions based on the assembled contigs, with BLASTN (version 2.2.13) against the NT and NR databases with E-value cutoff of 10^-5^. Among a total of 118,433 contigs, 26,332 of them (22.2%) were assigned to 356 taxa (genus), containing 330 bacterial genera and 26 archaeal genera. Comparable to the taxonomic structure generated from the output of BLAST based on reads, our analysis showed that Firmicutes (32.2%), followed by Proteobacteria (14.1%), Bacteroidetes (3.8%), and Euryarchaeota (5.5%), are most dominant (Figure 3B, Additional file 1: Table S1). The dominant classes in bacteria are Clostridia (8,249 contigs), Gammaproteobacteria (1,972 contigs), Bacilli (484 contigs), Bacteroidetes (315 contigs), and those in archaea were mapped to Methanomicrobia (1,279 contigs). The 17 most prevalent genera and the 6 most prevalent species are also consistent with the taxonomic structure based on mapped reads (Figure 3C, Additional file 1: Table S1).

Since 16S rDNA is widely used for taxonomic and phylogenetic studies due to its highly conserved sequences in both bacteria and archaea and its hypervariable region can also be used for accurate taxonomic evaluation, we extracted 793 contigs (only 0.7% of total contigs) that contain 16S rDNA sequences (an average length of 1,068 bp) for further analysis. When submitted to the RDP database (with 80% confidence), approximately 68.6% and 1.3% of them were classified into bacteria and archaea, respectively. At the class level, the dominant taxa include Clostridia, Anaerolineae, Synergistia, Methanomicrobia, Bacilli, and Gammaproteobacteria (Additional file 1: Table S2), mostly from Firmicutes, Proteobacteria and Euryarchaeota. It is noteworthy that the detection of classes Anaerolineae and Synergistia to be the dominant taxa based on 16S rDNA sequences differs from those based on reads and contigs. The reasons are more complex. One of them may be information loss in short contigs and sequence assemblies of low-abundance species that are difficult to annotate based on limited matches. Another may be due to the lower matching rate for the 16S-associated contigs, where only approximately 7% of the contigs were classified at the genus level. Therefore, an even lower number of 16S rDNA genes was detected and assigned to the profile with significant certainty.

### Novel *anaerococcus* and *psychrobacter* and their characteristics

We identified abundant reads assigned to two novel bacterial genera in the fermentation, and among these, the genus *Anaerococcus* of class Clostridia was identified, which was represented by the second most abundant bacterial species, *A*. *prevotii DSM 20548* (also as *A*. *prevotii* PC1, *Peptococcus prevotii*) (6,507 reads, 2.7%), an obligate anaerobic bacterium. *A*. *prevotii* often presents in oral cavity, skin, vagina, gut [31], and deep-seated soft tissue, causing abscesses or anaerobic infections in humans [32]. Thus far, in *Anaerococcus*, only this strain has been completely sequenced due to its clinical significance, and the hit-reads for *Anaerococcus* were all assigned to this strain. Therefore, at the species level, the taxonomic prediction should be treated with caution. Our taxonomic assignment depends on the comparison of amino acid sequences deduced from reads encoding protein sequences of known taxonomic origin. Therefore, only previously sequenced species can be identified and there are possibilities that other *Anaerococcus* species do exist in the fermenter but conformations from further studies are inevitable.

**Figure 5:**
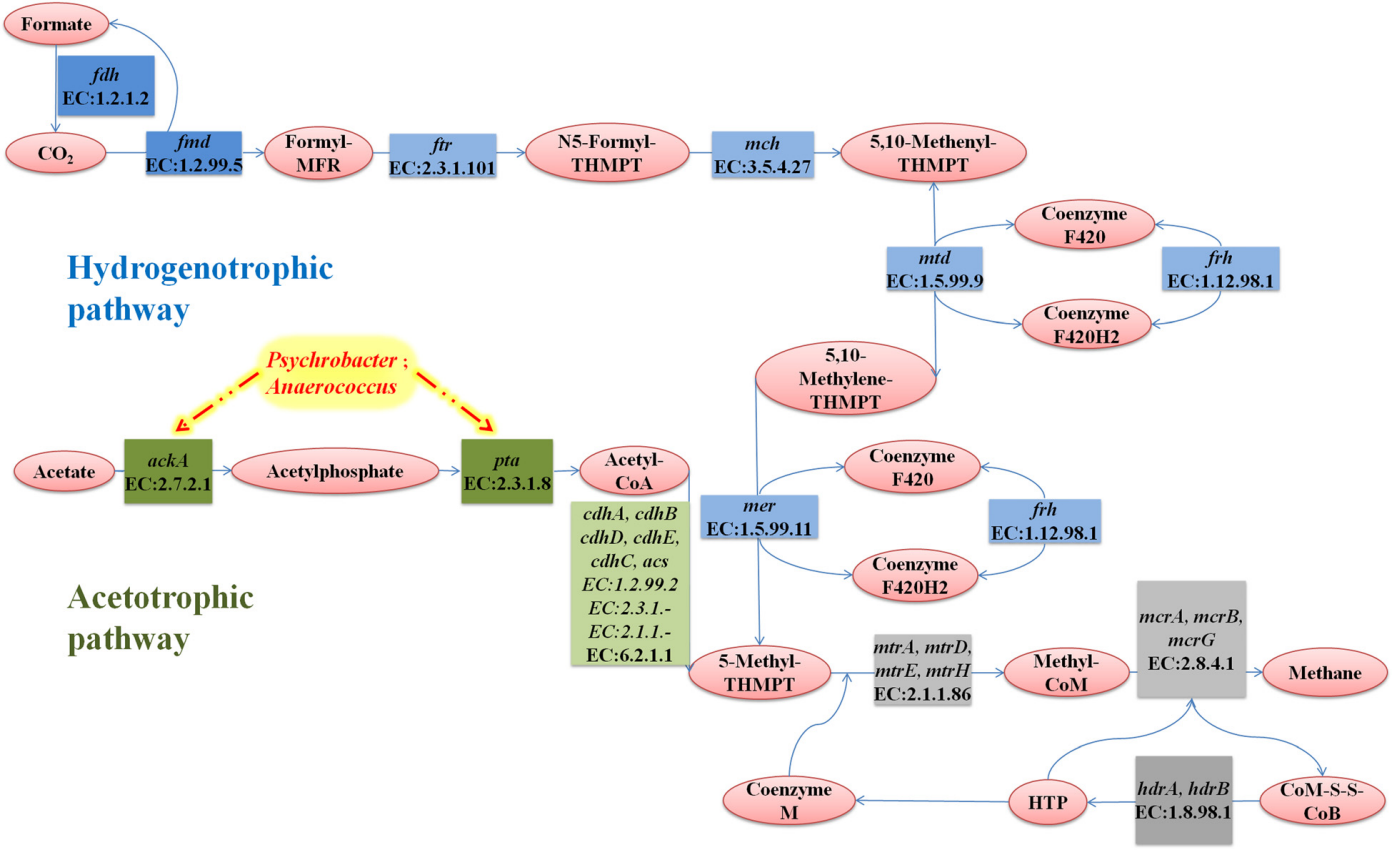
**Methanogenesis pathway predicted in our fermenter based on the KEGG analysis.** The ellipses denote the substances involved in the reaction. The boxes show the enzymes involved in methanogenesis. The acetotrophic (green), the hydrogenotrophic (blue), and the shared pathways (grey) are all color-encoded and the color depth indicates the amount of reads assigned. The enzymes that are potentially associated with *Anaerococcus* and *Psychrobacter* in the methanogenesis pathway are shown in the yellow box.

According to the literature, most species in *Anaerococcus* are capable of fermenting several carbohydrates, although the fermentation power is weak [33]. However, the involvement of abundant *Anaerococcus* species in anaerobic fermentation has never been reported. The function of abundant *Anaerococcus* species in the fermentation remains unknown. Its existence in the fermentation may be contaminations from kitchen wastes or variability of the genus itself; although genetically identical, the species possess significant discrepancies and are subject to adaptation under certain fermentation conditions. In this study, several important enzymes in the metabolic pathways for methane synthesis were detected in association with *Anaerococcus* based on the results of the KEGG analysis (Figure 5, Additional file 1: Table S3), such as ackA (acetate kinase, EC: 2.7.2.1), pta (phosphate acetyltransferase, EC: 2.3.1.8) and transporters/antiporters, including NhaC and V-type ATPase subunit D (EC: 3.6.3.14), indicating that *Anaerococcus* species likely contribute to the methane synthesis in the fermentation. Therefore, it is necessary to isolate *Anaerococcus* species, to characterize their phylogenetic relationships, and to study their biological and ecological functions in SS-AD and methanogenesis from SW in future studies.

The genus *Psychrobacter* (7,823 reads, 3.2%) of class Gammaproteobacteria (phylum Proteobacteria) is primarily comprised of *P*. *cryohalolentis* (1,431 reads, 0.6%), *P*. *arcticu*s (425 reads, 0.2%), and *Psychrobacter sp*. *PRwf*-*1* (524 reads, 0.2%). Most species of this genus can adapt to cold conditions, such as polar permafrost and ice, and are capable of reproducing at temperatures ranging from −10°C to 40°C [34]. The species can even be found in the relatively thermophilic environment of the digestion conditions. So far, *Psychrobacter* sp. have been defined as aerobic and mesophilic bacteria. However, some researchers have shown that some of their strains were also able to grow in fermenting environments [35-37] and anaerobic conditions, such as facultative anaerobic bacteria [38]. The *Psychrobacter* species often produce variable lipases (including phenylalanine deaminase, alkaline phosphatase, esterase (C4), esterase lipase (C8), lipase (C14), leucine arylamidase, and lecithinase [39,40]) and play essential roles in fat decomposition reactions. They have been isolated from the facial and body tissues of animals, poultry carcass, fermented seafood [38,41-43], and groundwater, but the isolation of *Psychrobacter* species has never been reported in biogas fermentation samples. In this study, several important enzymes in methane metabolism pathways associated with this genus were also detected based on the KEGG analyses(Figure 5, Additional file 1: Table S3), including ackA (acetate kinase, EC: 2.7.2.1), pta (phosphate acetyltransferase, EC: 2.3.1.8), acetyl-CoA synthetase (EC: 6.2.1.1) and transporters/antiporters (such as NhaA and NhaC), and the results indicate that these enzymes most likely participate in fat hydrolysis in SW samples for methanogenesis, particularly under mesophilic conditions.

Fat hydrolysis is the primary reaction of lipases. Moreover, lipases catalyze esterification, interesterification, acidolysis, alcoholysis and aminolysis reactions in addition to the hydrolytic activity on triglycerides [44]. Therefore, cold-active lipases, largely distributed in psychrophilic microorganisms and showing high catalytic activity at low temperatures, are added to detergents for cold washing, industrial food fermentation samples, environmental bioremediation (digesters, composting, oil or xenobiotic biology applications) and biotransformation processes [44]. Some cold-adaptive lipase genes from *Psychrobacter* sp. had been previously cloned and expressed [45]. Therefore, the abundance of *Psychrobacter* sp. in this fermenter demonstrates great potential for use in SW treatment for methane production or in other bio-energy conversion processes based on fatty-rich substrates, particularly under low temperature conditions, in northern China.

### Other dominant bacterial species and their characteristics

The most frequently predicted species in this fermenter is *A*. *colombiense DSM 12261* (11,868 reads, 5.1%), primarily isolated from anaerobic sludge and belongs to genus *Aminobacterium* (Clostridia) [46]. *A*. *colombiense DSM 12261* and the relative species of *Aminobacterium* are both syntrophic, capable of anaerobic degradation of amino acids, particularly without saccharides and consistently identified in anaerobic environment, such as sludge and compost [46,9]. The abundance of such amino acid-metabolizing organisms indicates high protein content in the SW samples.

*S*. *wolfei subsp*. *Wolfei str*. *Goettingen* (4,685 reads, 1.9%) belongs to genus *Syntrophomonas* (Clostridia), often isolated from anaerobic environments, such as aquatic sediment or sewage sludge, growing together with methanogens (such as *Methanospirillum hungatii*) and other H_2_-using and/or formate-using microorganisms [47]. *S*. *wolfei subsp*. *Wolfei str*. *Goettingen* participates in anaerobic fatty acid degradation [48] through the degradation of long-chain fatty acids into acetate and H_2_[49] due to the activity of acyl-CoA dehydrogenase, CoA transferase, enoyl-CoA hydratase, and other related enzymes [48], and this species plays a significant role in fatty acid decomposition and methanogenesis.

The reads for genus *Bacteroides* (5,655 reads, 2.1%) were mainly assigned to *B*. *thetaiotaomicron* VPI-5482 (1,406 reads, 0.6%), *B*. *vulgatus* ATCC 8482 (1,402 reads, 0.6%), and *B*. *fragilis* YCH46 (183 reads, 0.1%). *Bacteroides* often reside in human and animal intestines so that they exhibit symbiotic relationship with *E*. *coli* and other species. *Bacteroides* are involved in the fermentation of dietary polysaccharides, utilization of nitrogenous substances, and biotransformation of bile acids and other steroids in human colon [50]. Most intestinal bacteria are saccharolytic and obtain carbon and energy through hydrolysis of carbohydrates.

The dominant *Clostridium* species was identified as *C*. *thermocellum* (4,204 reads, 1.7%), which directly converts cellulosic substrate into ethanol with high efficiency and is a good candidate for the degradation of cellulosic materials from plant biomasses [20,51-53]. In addition to plants, some *Clostridium* species can also be isolated from animal feces and cultured with *Methanobacterium thermoautotrophicum*[51]. The tetanus-causing bacterium *C*. *tetani* (1,378 reads, 0.6%) is an obligate anaerobe that relies on fermentation. It can be found in manure-treated soil, animal feces, and fermentation samples from biogas-producing plant [20]. *C*. *kluyveri* (1,100 reads. 0.5%) grows anaerobically, using ethanol and acetate as sole energy sources to produce butyrate, caproate, and H_2_[54]. *C*. *kluyveri* is originally identified from canal mud [55] and *C*. *phytofermentans* (607 reads, 0.3%) is widely distributed in soil, capable of producing ethanol, acetate, CO_2_, and H_2_ through fermenting cellulose [56]. Therefore, in the initial steps of biomass digestion, members of *Clostridium* produce a wide variety of extracellular enzymes to degrade large biological molecules (such as cellulose, xylans, proteins, and lipids into fermentable components) [6] and participate in acetogenesis—the pathway prior to methanogenesis—to create precursors for methane production [17].

**Figure 6:**
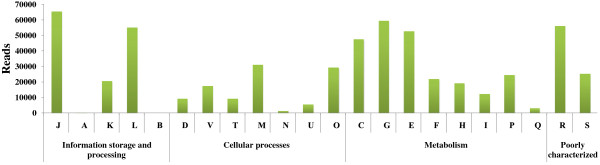
**Categorization of the biogas-fermenter metagenomic sequencing reads according to the Clusters of Orthologous Groups of proteins (COGs).** The categories are abbreviated as follows: **J**, translation, ribosomal structure and biogenesis; **A**, RNA processing and modification; **K**, transcription; **L**, replication, recombination and repair; **B**, chromatin structure and dynamics; **D**, cell cycle control, cell division, chromosome partitioning; **V**, defense mechanisms; **T**, signal transduction mechanisms; **M**, cell wall/membrane/envelope biogenesis; **N**, cell motility; **W**, extracellular structures; **U**, intracellular trafficking, secretion, and vesicular transport; **O**, posttranslational modification, protein turnover, chaperones; **C**, energy production and conversion; **G**, carbohydrate transport and metabolism; **E**, amino acid transport and metabolism; **F**, nucleotide transport and metabolism; **H**, coenzyme transport and metabolism; **I**, lipid transport and metabolism; **P**, inorganic ion transport and metabolism; **Q**, secondary metabolites biosynthesis, transport and catabolism; **R**, general function prediction only; and **S**, function unknown.

*Thermanaerovibrio acidaminovorans* DSM 6589 (1,948 reads, 0.8%) (also known as strain Su883) is a thermophilic anaerobic but air-tolerance organism [57]. This species is versatile, grows on a variety of amino acids, and can be co-cultured with *Methanobacterium thermoautotrophicum* Z245 to improve methane production [58]. The presence of this species in the sample suggests amino acid accumulation through protein and polypeptide degradation, and it is also evidenced by the fact that there are many genes assigned to the category of “amino acid transport and metabolism” in the COG and KEGG annotations (Figure 6).

### Dominant archaeal species and their characteristics

The most prevalent archaeal species was assigned to *Methanosarcina barkeri fusaro* (2,611 reads, 1.1%), which is originally isolated from sediment obtained from Lago del Fusaro, a freshwater coastal lagoon of West Naples, Italy [59]. This reference species possesses a relatively thick cell wall (composed of acidic heteropolysaccharide) that forms a protective sheath, and it uses versatile substrates for methane synthesis, making it more adaptable to the environment as compared with its relatives. In addition to its strong survival ability, *M*. *barkeri* is capable of raising pH level in the surrounding area [60]. Similar to landfill, solid-state fermentation also accumulates acids produced by acetogens that make the environment too acidic to host methanogens. However, attributable to the *M*. *barkeri* accumulation, a lesser acidic environment can be maintained in the fermenter and other methanogens can benefit from it. In addition, this microbe often makes trash mound more compact and creates more room for waste treatment [60].

The genus *Methanosarcina* includes many methanogens whose metabolic features are diverse and include both acetotrophic and hydrogenotrophic pathways. In particular, some strains in this genus are capable of utilizing methanol [59]. Furthermore, most *Methanosarcina* are immotile and mesophilic, exhibiting multiple metabolic features with strong advantage in survival. It is proposed that methanol is one of the major factors that influence methanogenesis [61]. In SW treatments, there are approximately 60% of the total mass containing complex organic matters and products of hydrolysis and acidogenesis are most likely multiple since members of order Methanosarcinales have the widest substrate range among methanogens [62]. The dominance of *Methanosarcina* demonstrates the relatively abundant nutrient sources and various metabolic pathways within our fermentation system.

The second dominant archaeal taxon is *Methanoculleus marisinigri* JR1 (1,101 reads, 0.5%), an organism that belongs to order Methanomirobiales and class Methanomicrobia. This species is capable of producing methane through the reduction of CO_2_ with H_2_ and uses formate and secondary alcohols as alternative electron donors sometimes, i.e., the hydrogenotrophic pathway. However, *M*. *marisinigri* JR1 cannot use acetate and methyl group-containing compounds for methanogenesis, i.e., the acetotrophic pathway. *M*. *marisinigri* JR1 is relatively small in cell dimensions and grows under moderate conditions with temperature ranging from 10°C to 32°C and pH ranging from 6.8 to 7.3. *M*. *marisinigri* JR1 is found in both thermophilic anaerobic digester [63] and the leachate of a full-scale recirculating landfill [64]. Particularly, in a metagenomic study of methanogens residing in a biogas-producing plant in Germany, *M*. *marisinigri* JR1 is found being the most abundant species in the reactor [20].

The third abundant archaeal methanogen was identified as *Methanosaeta theromphila* (724 reads, 0.3%), a member of *Methanosaeta*, which is the only genus of family Methanosaetaceae. *M*. *theromphila* are non-motile, non-sporulating, and thermophilic, which thrives at temperature of 50°C or higher, though it only grows at near neutral pH. *M*. *theromphila* is rod-shaped and capable of producing acetate kinase that activates acetate to acetyl-CoA in the first step of fermenting acetate to methane [65].

### Gene function annotation and classification

To obtain a metabolic profile for this discrete bacterial community, we annotated all sequences (total reads) using BLASTX based on COG and KEGG database (Figure 6). Approximately 28% of the total reads were assigned to one or more COG functional categories. In the category “metabolism”, a large amount of reads are distributed among “carbohydrate transport and metabolism (G)”, “amino acid transport and metabolism (E)”, “energy production and conversion (C)” and “lipid transport and metabolism (I)” (Additional file 1: Figure S3). These metabolic activities are associated with the conversion of biomass into methane during anaerobic fermentation. In the KEGG analysis, metabolism terms, including purine, pyrimidine, amino sugar and nucleotide sugar, glycolysis/gluconeogenesis and methane metabolisms are among the top five most popular categories (Additional file 1: Figure S4). Many of these metabolic processes are involved in the conversion of carbohydrates to simple compounds and the use of methane in the absence of oxygen. For example, pyruvate:ferredoxin oxidoreductase and related 2-oxoacid:ferredoxin oxidoreductases (COG0674, 2217 reads), Glycosidases (COG0366, 2632 reads), nucleoside-diphosphate-sugar epimerases (COG0451, 2219 reads), sugar permeases (COG0395, 2050 reads), and glucan phosphorylase (COG0058, 1211 reads) were all inevitably detected in this system.

The enzymes involved in carbohydrate metabolism were detected in reads assigned to “amino and nucleotide sugar metabolisms (4,217 reads)”, “glycolysis/gluconeogenesis (4,212 reads)” and “starch and sucrose metabolisms (3,170 reads)” as the three most dominant groups, which are involved in processing of monosaccharides and polyose, such as maltase-glucoamylase [EC: 3.2.1.20], beta-glucosidase [EC: 3.2.1.21], glycogen-debranching enzyme [EC: 2.4.1.25 3.2.1.33], levansucrase [EC: 2.4.1.10], chitinase [EC: 3.2.1.14], and glucokinase [EC: 2.7.1.2]. This observation is consistent with the finding that many species in this fermentation sample are involved in carbohydrate digestion and energy conversion.

There are also abundant reads that matched to genes for “lipid metabolism” (4,385 reads), such as fatty acid, glycerolipid, glyceropholipid, arachidonic acid, and linoleic acid metabolisms. Many of the enzymes detected in the processes, such as dihydroxyacetone kinase [EC: 2.7.1.29], glycerate kinase [EC: 2.7.1.31], glycerol-3-phosphate dehydrogenase (NAD(P)+) [EC: 1.1.1.94], glycerol-3-phosphate dehydrogenase [EC: 1.1.5.3] and acetyl-CoA acyltransferase [EC: 2.3.1.16], are also involved in methane metabolism. In addition, a significant amount of reads were obtained for the processes involved in the protein degradation pathway (1,820 reads), such as ATP-dependent Clp protease proteolytic subunit [EC: 3.4.21.92] and ATP-dependent protease La [EC: 3.4.21.53], EC: ATP-dependent protease HsIV [EC: 3.4.25]. Approximately 15% (v/v) of the kitchen waste in our fermenter contain both fat and protein, and both lipid hydrolysis and peptide degradation provide fermentation substrates for the downstream methanogenesis.

Moreover, total contigs with lengths longer than 500 bp were also analyzed against the KEGG database based on the BLAST tools. Non-eukaryotic contigs ranging from 10 to 60 kbp were extracted from the BLAST output files, and the contigs with identities lower than 80% or with alignment lengths shorter than 100 bp were filtered out. For each contig, we selected the best-hit sequences based on the highest score. The functional annotations of the large contigs (Additional file 1: Table S4) showed that there are 16 contigs with hits to genetic information processing pathway, 12 contigs for environmental information processing, 9 contigs for cellular processes, and 8 contigs for nucleotide metabolism. For amino acid, carbohydrate, and energy metabolism, the numbers of large contigs with best-hits were 4, 4, and 3, respectively. Larger contig suggests higher sequencing-read coverage. Therefore, the abundant microorganisms are always the active participants in the degradation of organic materials and energy exchange under the fermentation conditions.

### Metabolic pathway analysis in the SW fermentation

The two distinct methanogenic pathways are from H_2_/CO_2_ to methane (hydrogenotrophic pathway) and from acetate to methane (acetotrophic pathway). Methanogenesis has also been shown to use carbon from other small organic compounds, such as formate, methanol, methylamines, dimethyl sulfide, and methanethiol, which are usually classified intermediates or substrates of the H_2_/CO_2_-to-methane pathway. Figure 5 shows the elements of the two methanogenesis pathways detected in our study. Many large contigs (20 contigs in a total length of 76,331 bp; Additional file 1: Table S3), such as contig17513 (10,585 bp) for the formate dehydrogenase (EC: 1.2.1.2) and contig06034 (10,805 bp) for the formylmethanofuran dehydrogenase (fwdA/fmdA, EC: 1.2.99.5), which are involved in the initial step of the hydrogenotrophic pathway (Figure 5, blue box), were detected in the sample. In addition, *mtd*, *mer* (EC: 1.5.99.9), *frhB* (EC: 1.12.98.1), *ftr* (EC: 2.3.1.101) and *mch* (EC: 3.5.4.27), which are also involved in the hydrogenotrophic pathway, were present. Moreover, a significant number of contigs (17 contigs, total length 38,078 bp, in Additional file 1: Table S3) were mapped to the acetotrophic pathway (Figure 5, green boxes). In this pathway, acetyl-CoA synthetase (*acs*, EC: 6.2.1.1) plays a key role in the synthesis of acetyl-CoA from acetate. Acetyl-CoA synthetase is involved in the acetyl-CoA decarbonylase/synthase complex (ACDS; composed of CdhA1, CdhB, CdhD, CdhE and CdhC) and cleaves C-C/C-S bonds in the acetyl moiety of acetyl-CoA to oxidize the carbonyl group into CO_2_ and to transfer the methyl group to tetrahydrosarcinapterin.

Methanogens use 2-mercaptoethanesulfonate (CoM; coenzyme M) as the terminal methyl carrier in methanogenesis. Tetrahydromethanopterin S-methyltransferase (*mtr*, EC: 2.1.1.86), methyl coenzyme M reductase (*mcr*, EC: 2.8.4.1) and reductase heterodisulfide reductase (*Hdr*, EC: 1.8.98.1), which are required for the final reaction steps of both methanogenic pathway, were also identified in our sample (Figure 5 and Additional file 1: Table S3). Furthermore, the finding of critical enzymes, such as phosphosulfolactate synthasein (*coma*, EC: 4.4.1.19), 2-phosphosulfolactate phosphatase (*comb*, EC: 3.1.3.71), and (R)-2-hydroxyacid dehydrogenase (EC: 1.1.1.272), for coenzyme M biosynthesis (data not shown in Figure 5 but in Additional file 1: Table S3) provides insights into the SW fermentation process. Moreover, our pathway analyses defined a variety of transporters/antiporters involved in the methanogenic pathways, such V-type H + −transporting ATPase and Na+:H + antiporter (nha) (not shown in Figure 5 but in Additional file 1: Table S3). Therefore, both hydrogenotrophic and acetotrophic pathways for methanogenesis occur almost equally in our fermenter, and the conclusion is strongly supported by the evidence from our data and consistent with the metabolic characteristics of the dominant archaeal species and complex components of the microbial communities in the SW fermentation.

## Conclusions

Using high-throughput pyrosequencing and optimized DNA extraction protocols, we characterized microbial communities of mesophilic SS-AD fermentation and their related metabolic pathways in biomass degradation and methane synthesis. First, we aligned the reads and assembled contigs separately to the related databases and found that bacteria and archaea took 91.5% and 4.4% of the hits from the sequencing reads, respectively. Members from Firmicutes, Clostridia and Bacilli, are mostly enriched, followed by phyla Proteobacteria and Bacteroidetes. Particularly, the species from genera *Aminobacterium*, *Psychrobacter*, *Anaerococcus*, *Clostridium*, *Syntrophomonas*, and *Bacteroides* play key roles in the initial degradation of protein, fat, cellulose, and other polysaccharides. These results were further supported by gene functional annotation where we detected many enzymes involved in “protein degradation”, “lipid metabolism”, and “carbohydrate metabolism”.

Second, the dominant methanogens present in this fermenter were from Methanomicrobia. The most prevalent species appears to be *Methanosarcina barkeri fusaro*, which uses versatile substrates and contains both acetotrophic and hydrogenotrophic pathways for methane synthesis [62]. *M*. *marisinigri* JR1 and *M*. *theromphila* with either hydrogenotrophic or acetotrophic pathways for methanogenesis appear less abundant.

Third, the *Psychrobacter* (class Gammaproteobacteria) and *Anaerococcus* (class Clostridia) species are obviously abundant in the fermenter, but they have seldom been reported in other biogas fermentation samples. The *Psychrobacter* species adapt to extremely cold climates and produce cold-adaptive lipases [34] and have great potential to be used in low-temperature fermentation, particularly in northern China. However, *Anaerococcus* species exhibit weak fermentation capability [33] but abundant in SS-AD, playing roles in biomass degradation efficiency and methane yield. Our findings indicate that it is important to identify these species and to characterize them for their ecological and biological functions under SS-AD conditions, particularly for the rational design of microbial community structures to improve biogas production in solid-state fermentation under low-temperature conditions.

## Methods

### Sample preparation for DNA extraction

The samples for total DNA extraction were obtained from an anaerobic digester with a 2-liter working capacity. The digester was loaded with multi-component substrates, including kitchen waste (15%, v/v over the total solid added), pig manure (42.5%) and excess sludge (42.5%), and the initial total solid content was 20% (v/v for the total container volume). The anaerobic digestion was operated at 35 ± 1°C. The samples were collected from the digester when biogas production entered a steady phase. On the sampling day, the biogas yield was 72% biomethane at pH 7.0.

### Total DNA extraction

The liquid content of samples (0.25 g fresh weight) was removed by centrifugation at 13,000 rounds per minute (rpm) for 10 min at 4°C. Subsequently, five different protocols (Protocols E, EY, F, P, and S) were used to extract total DNA according to the manufacturer’s instructions and laboratory manuals. 30-μl double distilled (dd) H_2_O were used to dissolve the DNA at the final step regardless what stated in the various protocols.

Protocol E: the E.Z.N.A.TM Soil DNA Kit (Omega Bio-Tek, Inc., USA) was used with minor modifications. Briefly, in the lysis step, vortexing was replaced by hand shaking for approximately 10 min to dissolve the pellet.

Protocol EY: The sample (0.25 g) was washed twice with 1.5 ml of TENP buffer [66], vortexed for 10 min, collected through centrifugation (12000 rpm, 5 min), neutralized with 1 ml of PBS buffer, and subjected to Protocol E for DNA extraction.

Protocol F: the FastDNA Spin Kit (for soil DNA extraction, MP Biomedicals, Heidelberg, Germany) was used with small adjustment in the lysis step as in Protocol E. In the purification step, the Spin Filter was washed twice with 500 μl SEWS-M buffer for better DNA purity.

Protocol P: the Mo-Bio PowerSoil DNA Isolation Kit (MoBio Laboratories, Carlsbad, CA, USA) was used with minor modifications. The original lysis time was changed to 15 min with maximum intensity, and the sample was centrifuged for longer time (12000 rpm, 2 min) to completely degrade cell walls. In the purification step, the Spin Filter was washed twice with 500 μl of solution C5 for better DNA purity.

Protocol S: the sample (0.25 g) was pre-washed as done in Protocol EY before DNA extraction according to modified method of Zhou et al. (1996) [67]. Briefly, after adding 0.25 g glass beads (d = 1 mm) and 0.75 ml DNA extraction buffer (100 mM Tris, 100 mM EDTA, 200 mM NaCl, 0.01 g/ml PVP, 2% CTAB, pH = 8.0) to the pretreated pellet, the sample was vortexed for 5 min. Subsequently, 0.75 ml SDS buffer (100 mM Tris, 200 mM NaCl, 2% SDS, pH = 8) was added and mixed with hand-shaking for 5 min. The sample was incubated at 65°C for 10 min and inverted every 10 min for a total of 5 times. After centrifugation at 12000 rpm for 15 min at room temperature, the middle-layer liquid was collected, extracted with an equal volume of chloroform-isoamyl alcohol (24:1, v/v), precipitated with isopropanol, and washed with 70% ethanol.

### DNA quantification

The total DNA yield and quality were determined spectrophotometrically (NanoDrop 3300, Thermo Fisher Scientific Inc. USA), followed by electrophoresis on 0.8% agarose gels.

### T-RFLP analysis

The 16S rDNA was PCR amplified using the universal bacterial primer set containing 8 F-FAM (5^′^-AGAGTTTGATCMTGGCTCAG-3^′^) and 1492R (5’- GGTTACCTTGTTACGACTT-3^′^) [68] and the archaeal domain-specific primer set containing Arc109F-FAM (5^′^-ACKGCTCAGTAACACGT-3^′^) and Arc 915R (5^′^-GTGCTCCCCCGCCAATTCCT-3^′^) [69], respectively. The 5^′^-ends of primers 8 F and Arc109F were labeled with 6-carboxyfluoresceinphosphoramidite (FAM). The PCR reactions were performed with an rTaq-polymerase (TAKARA biotechnology (Dalian) Co., Ltd., Japan.) for 25 cycles and the annealing temperature was 60°C for bacteria and 55°C for archaea. The PCR products were subsequently purified using the QIAquick PCR purification kit (QIAGEN China Co., Ltd., Germany), and a 50-μl aliquot of each PCR product was digested with the restriction enzymes *Msp*I and *Taq*I (New England Biolabs (Beijing) Co., Ltd. USA), respectively, for 2 h and subjected to the gene scan analysis on an ABI 3730 DNA Analyzer at Shanghai GeneCore BioTechnologies Co., Ltd. China prior to terminal restriction fragment length polymorphism (T-RFLP) analysis.

### Pyrosequencing of total DNA

Total DNA from fermentation samples was sheared and sized to produce DNA whole-genome-shotgun library according to the manufacturer’s protocol from GS FLX Titanium General Library Preparation Kit (Roche Applied Science, USA). DNA Sequencing was performed on a 454 GS FLX Titanium platform at the Beijing Institute of Genomics, Chinese Academy of Sciences.

### Statistics of the biogas-metagenome sequencing data

The shotgun sequences were assembled by using the GS de novo assembler. Raw and statistical sequencing data were summarized according to the assembly output. Both raw reads and contigs were used for further analysis.

### Classification of sequencing data

The classification of the total data was performed by using the BLASTN/BLASTX tools against GenBank NT/NR databases with an E-value cutoff of 10^-5^ based on total reads and contigs.

The species richness analysis was performed by using MEGAN based on total sequencing reads [70]. The MEGAN platform uses the lowest common ancestor (LCA) algorithm to classify reads to certain taxa based on their blast hits. The LCA parameters were set as Min Score 35.0, Top Percent 50, and Min Support 2.

In addition, the 16S rDNA contigs with hits were extracted from the results of BLASTN analysis against the NT database and submitted to the Ribosomal Database Project (RDP) database [71] for classification with 80% confidence.

A rarefaction curve was generated for all reads, except unassigned and no-hit reads. The results of the total read classification were constructed into a rooted taxonomic tree where each clade (leaf) represents a taxon. The clades (leaves) in this tree were subsequently used as operational taxonomic units (OTUs) in the rarefaction analysis. The program randomly and incrementally chooses a tenth of the reads as a subset until all the reads are chosen. For each random subset, the number of leaves is determined independently.

### Functional annotation of total contigs

To obtain gene profile characteristic for the anaerobic microbial community, the total sequencing reads were annotated based on BLASTX analysis against the database of Clusters of Orthologous Groups of proteins (COG) [72] with an *E*-value cut-off of 10^−5^. The sequencing reads were functionally annotated and assigned to the COG categories according to their best hits.

The metabolism analysis was performed on KEGG Orthology (KO)-identifiers by using KAAS tool (KEGG Automatic Annotation Server) with bi-directional best hit of total contigs, a default threshold (60), and prokaryotes as a representative set. Gene annotation was based on Enzyme Commission (EC)-numbers based on the Kyoto Encyclopedia of Genes and Genomes (KEGG) Orthology database. Metabolic pathway maps were drawn according to the list of unique EC numbers.

## Abbreviations

SS-AD: Solid state anaerobic digestion;SW: Solid biomass waste;PBS buffer: Phosphate buffered saline;CTAB: Cetyltrimethylammonium bromide;EDTA: Ethylene diamine tetraacetie acid;PVP: Polyvinylpyrrolidone;SDS: Sodium dodecyl sulfate;rpm: Rounds per minute;FAM: 6-Carboxyfluoresceinphosphoramidite;T-RFLP: Terminal restriction fragment length polymorphism;nt: Nucleotide;NT database: Non-redundant nucleotide sequence database, with entries from all traditional divisions of GenBank EMBL, and DDBJ excluding bulk divisions (gss, sts, pat, est, htg divisions) and wgs entries;NR database: Non-redundant protein sequence database with entries from GenPept, swissprot, PIR, PDF, PDB, and RefSeq;LCA: Lowest common ancestor;RDP database: Ribosomal database project database;OTUs: Operational taxonomic units;COG: Clusters of orthologous groups of proteins;KEEG: Kyoto encyclopedia of genes and genomes orthology database;KO: KEGG ortholog;KAAS: KEGG automatic annotation server;EC: Enzyme commission;dd H2O: Double distilled H_2_O;CoM: Coenzyme M;CoA: Coenzyme A;ACDS: Acetyl-CoA decarbonylase/synthase complex;NAD(P)+: Nicotinamide adenine dinucleotide (phosphate);16S rDNA: 16S rRNA gene sequence;PCR: Polymerase chain reaction

## Competing interest

The authors declare that were no competing interests.

## Authors’ contributions

Study concept and design: SL, JY. Fermentation setting and sampling: AL, LZ. DNA extraction and evaluation: AL, YC. Bioinformatics analysis: YC, AL. Analysis and interpretation of data: YC, AL. Acquisition of data: SW, XW, LR, JY. Drafting of the manuscript: AL, YC, SW. Critical revision of the manuscript for important intellectual content: SL JY. Obtaining funding: SL, JY. Administrative, technical, or material support: XW, LR, SW, XL. Supervision: SL, JY. All the authors have read and approved the final manuscript.

## Authors’ information

S Li is a Professor, A Li is a M. Phil student, X Liu is an Associate Professor, and J Yan and L Zhang are postdocs of Institute of Nuclear and New Energy Technology, Tsinghua University, China.

J Yu is a Professor of the CAS Key Laboratory of Genome Sciences and Information, Beijing Institute of Genomics (BIG), Chinese Academy of Sciences (CAS), China. Y Chu is a PhD student, and X Wang, L Ren and S Wu are Associate Professors of BIG, CAS, China.

All abbreviations for enzymes and genes involved in the methanogenesis pathways in Figure 5 are listed in Additional file 1: Table S3.

## Supplementary Material

Additional file 1: Table S1 Top 17 genera of taxonomic classification based on contig-counts. **Table S2.** Analysis of bacterial and archaeal 16S-rDNA contigs based on the Ribosomal Database Project Classifier (RDPC). **Table S3.** Large contig function annotation. **Table S4.** The list of contigs detected in methanogenesis pathways. **Figure S1.** The histogram shows the distribution of the GC percentage for BE-1 sample. Each position represents the number of sequences within a GC percentage range. The data used in these graphs is based on raw upload and post quality-control sequences. **Figure S2.** Comparison of microbial community structures between BE-1 and BEY. The taxonomic trees of BE-1 and BEY on rank family for archaea and on rank class for bacteria were constructed respectively on MEGAN. A, archaea of BE-1. B, archaea of BEY. C, bacteria of BE-1. D, bacteria of BEY. **Figure S3.** Popular terms in metabolism based on KEGG analysis. The Y-axis refers to the percentage of reads within the reads mapping to metabolism terms. **Figure S4.** Popular terms in the functional secondary category of metabolism based on KEGG analysis. (DOC 1278 kb) Click here for file
