# Supplementary material for: A pyrosequencing-based metagenomic study of methane-producing microbial community in solid-state biogas reactor
Source: Biotechnol Biofuels. 2013 Jan 15;6:3. doi: 10.1186/1754-6834-6-3 (PMC3618299; doi:10.1186/1754-6834-6-3)
Supplement: Additional file 1: Table S1 — Top 17 genera of taxonomic classification based on contig-counts. Table S2. Analysis of bacterial and archaeal 16S-rDNA contigs based on the Ribosomal Database Project Classifier (RDPC). Table S3. Large contig function annotation. Table S4. The list of contigs detected in methanogenesis pathways. Figure S1. The histogram shows the distribution of the GC percentage for BE-1 sample. Each position represents the number of sequences within a GC percentage range. The data used in these graphs is based on raw upload and post quality-control sequences. Figure S2. Comparison of microbial community structures between BE-1 and BEY. The taxonomic trees of BE-1 and BEY on rank family for archaea and on rank class for bacteria were constructed respectively on MEGAN. A, archaea of BE-1. B, archaea of BEY. C, bacteria of BE-1. D, bacteria of BEY. Figure S3. Popular terms in metabolism based on KEGG analysis. The Y-axis refers to the percentage of reads within the reads mapping to metabolism terms. Figure S4. Popular terms in the functional secondary category of metabolism based on KEGG analysis. (DOC 1278 kb) [file 1754-6834-6-3-S1.doc]

**Additional file Table Captions：**

**Additional file Table S1** Top 17 genera of taxonomic classification based on contig-counts.

**Additional file Table S2** Analysis of bacterial and archaeal 16S-rDNA contigs based on the Ribosomal Database Project Classiﬁer (RDPC).

**Additional file Table S3** The list of contigs detected in methanogenesis pathways.

**Additional file Table S4** Large contig function annotation.

**Additional file Figure Legends：**

**Additional file Figure S1** The histogram shows the distribution of the GC percentage for BE-1 sample. Each position represents the number of sequences within a GC percentage range. The data used in these graphs is based on raw upload and post quality-control sequences.

**Additional file Figure S2** Comparison of microbial community structures between BE-1 and BEY. The taxonomic trees of BE-1 and BEY on rank family for archaea and on rank class for bacteria were constructed respectively on MEGAN. A, archaea of BE-1. B, archaea of BEY. C, bacteria of BE-1. D, bacteria of BEY.

**Additional file Figure S3** Popular terms in metabolism based on KEGG analysis. The Y-axis refers to the percentage of reads within the reads mapping to metabolism terms.

**Additional file Figure S4** Popular terms in the functional secondary category of metabolism based on KEGG analysis

**Additional file Table S1**

| Phylum | Class | Order | Family | Genus | Counts of contigs assembled # |
| --- | --- | --- | --- | --- | --- |
| Firmicutes | Clostridia | Clostridiales | Clostridiaceae | ***Clostridium*** | 2028 |
|  | Clostridia | Clostridiales | Syntrophomonadaceae | ***Aminobacterium*** | 1769 |
|  | Clostridia | Clostridiales | Peptostreptococcaceae | ***Anaerococcus*** | 1038 |
|  | Clostridia | Clostridiales | Syntrophomonadaceae | ***Syntrophomonas*** | 528 |
|  | Clostridia | Clostridiales | Clostridiaceae | ***Alkaliphilus*** | 334 |
|  | Clostridia | Clostridiales | Syntrophomonadaceae | ***Thermanaerovibrio*** | 193 |
|  | Bacilli | Lactobacillales | Enterococcaceae | ***Enterococcus*** | 184 |
|  | Clostridia | Thermoanaerobacteriales | Thermoanaerobacteriaceae | ***Thermoanaerobacter*** | 154 |
|  | Bacilli | Lactobacillales | Streptococcaceae | ***Streptococcus*** | 152 |
|  | Bacilli | Bacillales | Bacillaceae | ***Bacillus*** | 148 |
|  |  |  |  |  |  |
| Bacteroidetes | Bacteroidetes | Bacteroidales | Bacteroidaceae | ***Bacteroides*** | 315 |
|  |  |  |  |  |  |
| Proteobacteria | Gammaproteobacteria | Pseudomonadales | Moraxellaceae | ***Psychrobacter*** | 1101 |
|  | Gammaproteobacteria | Pseudomonadales | Pseudomonadaceae | ***Pseudomonas*** | 236 |
|  | Gammaproteobacteria | Enterobacteriales | Enterobacteriaceae | ***Enterobacter*** | 84 |
|  |  |  |  |  |  |
| Euryarchaeota | Methanomicrobia | Methanosarcinales | Methanosarcinaceae | ***Methanosarcina*** | 905 |
|  | Methanomicrobia | Methanosarcinales | Methanosaetaceae | ***Methanosaeta*** | 164 |
|  | Methanomicrobia | Methanomirobiales | Methanomicrobiaceae | ***Methanoculleus*** | 95 |

# Counts of contigs matched to top 17 abundant microbial genera. Results were obtained from the best BLASTx hits by searching the nucleotide sequence database of NCBI GenBank.

**Additional file Table S2**

| Domain | Phylum | Class | No. of contigs hit 16s rDNA^#^ |
| --- | --- | --- | --- |
| Archaea | Euryarchaeota | *Methanomicrobia* | 6 |
|  |  | *Methanobacteria* | 3 |
| Bacteria | Firmicutes | *Clostridia* | 60 |
|  |  | *Bacilli* | 6 |
|  | Chloroflexi | *Anaerolineae* | 8 |
|  | Firmicutes | *Synergistia* | 7 |
|  | Proteobacteria | *Gammaproteobacteria* | 6 |
|  |  | *Alphaproteobacteria* | 1 |
|  |  | *Deltaproteobacteria* | 1 |
|  | Thermotogae | *Thermotogae* | 2 |
|  | Bacteroidetes | *Bacteroidia"* | 2 |
|  |  | *Flavobacteria* | 1 |
|  | Actinobacteria | *Actinobacteria* | 1 |
|  | Planctomycetes | *Planctomycetacia"* | 1 |

**^#^** Only assignments with conﬁdence values above 80% were considered for this chart.

|  |  |  |  |  |  |  |  |  |
| --- | --- | --- | --- | --- | --- | --- | --- | --- |
|  |  |  |  |  |  |  |  |  |
|  |  |  |  |  |  |  |  |  |
|  |  |  |  |  |  |  |  |  |
|  |  |  |  |  |  |  |  |  |
|  |  |  |  |  |  |  |  |  |
|  |  |  |  |  |  |  |  |  |
|  |  |  |  |  |  |  |  |  |
|  |  |  |  |  |  |  |  |  |
|  |  |  |  |  |  |  |  |  |
|  |  |  |  |  |  |  |  |  |
|  |  |  |  |  |  |  |  |  |
|  |  |  |  |  |  |  |  |  |
|  |  |  |  |  |  |  |  |  |
|  |  |  |  |  |  |  |  |  |
|  |  |  |  |  |  |  |  |  |
|  |  |  |  |  |  |  |  |  |
|  |  |  |  |  |  |  |  |  |
|  |  |  |  |  |  |  |  |  |
|  |  |  |  |  |  |  |  |  |
|  |  |  |  |  |  |  |  |  |
|  |  |  |  |  |  |  |  |  |
|  |  |  |  |  |  |  |  |  |
|  |  |  |  |  |  |  |  |  |
|  |  |  |  |  |  |  |  |  |
|  |  |  |  |  |  |  |  |  |
|  |  |  |  |  |  |  |  |  |
|  |  |  |  |  |  |  |  |  |
|  |  |  |  |  |  |  |  |  |
|  |  |  |  |  |  |  |  |  |
|  |  |  |  |  |  |  |  |  |
|  |  |  |  |  |  |  |  |  |
|  |  |  |  |  |  |  |  |  |
|  |  |  |  |  |  |  |  |  |
|  |  |  |  |  |  |  |  |  |
|  |  |  |  |  |  |  |  |  |
|  |  |  |  |  |  |  |  |  |
|  |  |  |  |  |  |  |  |  |
|  |  |  |  |  |  |  |  |  |
|  |  |  |  |  |  |  |  |  |
|  |  |  |  |  |  |  |  |  |
|  |  |  |  |  |  |  |  |  |
|  |  |  |  |  |  |  |  |  |
|  |  |  |  |  |  |  |  |  |
|  |  |  |  |  |  |  |  |  |
|  |  |  |  |  |  |  |  |  |
|  |  |  |  |  |  |  |  |  |
|  |  |  |  |  |  |  |  |  |
|  |  |  |  |  |  |  |  |  |

| **Additional file Table S3** | |  |  |  |  |  |
| --- | --- | --- | --- | --- | --- | --- |
| Pathway | EC number | KEGG ORTHOLOGY | Enzymes | KO number | Contig hit | Length (bp) |
| Coenzyme M biosynthesis | EC:1.1.1.272 |  | (R)-2-hydroxyacid dehydrogenase | K05884 | contig01110 | 6139 |
|  | EC:3.1.3.71 | comB | 2-phosphosulfolactate phosphatase | K05979 | contig68632 | 518 |
|  | EC:4.4.1.19 | comA | phosphosulfolactate synthase | K08097 | contig68860 | 828 |
|  |  |  | phosphosulfolactate synthase | K08097 | contig87780 | 399 |
| acetate => methane | EC:1.2.99.2 | cdhA | acetyl-CoA decarbonylase/synthase complex subunit alpha | K00192 | contig28970 | 668 |
|  | EC:2.3.1.- | cdhC | acetyl-CoA decarbonylase/synthase complex subunit beta | K00193 | contig23317 | 289 |
|  |  |  | acetyl-CoA decarbonylase/synthase complex subunit beta |  | contig97244 | 267 |
|  | EC:1.2.99.2 | cdhD | acetyl-CoA decarbonylase/synthase complex subunit delta | K00194 | contig46178 | 729 |
|  |  |  |  |  | contig77593 | 350 |
|  |  | cdhB | acetyl-CoA decarbonylase/synthase complex subunit epsilon | K00195 | contig48585 | 697 |
|  | EC:2.1.1.- | cdhE | acetyl-CoA decarbonylase/synthase complex subunit gamma | K00197 | contig73532 | 459 |
|  | EC:2.3.1.8 | pta | phosphate acetyltransferase | K00625 | contig101108 | 4497 |
|  |  |  |  |  | contig103159 | 4086 |
|  |  |  |  |  | contig33601 | 3729 |
|  | EC:2.7.2.1 | ackA | acetate kinase | K00925 | contig18053 | 2922 |
|  |  |  |  |  | contig41730 | 1601 |
|  | EC:6.2.1.1 | acs | acetyl-CoA synthetase | K01895 | contig08295 | 8457 |
|  |  |  |  |  | contig114002 | 6351 |
|  |  |  |  |  | contig33477 | 1509 |
|  |  |  |  |  | contig50160 | 823 |
|  |  |  |  |  | contig44927 | 644 |
| formate => methane | EC:1.2.1.2 |  | formate dehydrogenase, alpha subunit | K00123 | contig17513 | 10585 |
|  |  |  |  |  | contig15997 | 6644 |
|  |  |  |  |  | contig25620 | 3607 |
|  |  |  | formate dehydrogenase, beta subunit | K00124 | contig01145 | 28248 |
|  |  |  |  | K00125 | contig50150 | 429 |
|  |  |  | formate dehydrogenase, gamma subunit | K00127 | contig52008 | 942 |
|  | EC:1.2.99.5 | fwdA, fmdA | formylmethanofuran dehydrogenase subunit A | K00200 | contig06034 | 10805 |
|  |  | fwdC, fmdC | formylmethanofuran dehydrogenase subunit C | K00202 | contig36802 | 442 |
|  |  |  |  |  | contig73033 | 355 |
|  |  | fwdD, fmdD | formylmethanofuran dehydrogenase subunit D | K00203 | contig65074 | 399 |
|  |  | fmdF | formylmethanofuran dehydrogenase subunit F | K00205 | contig68481 | 553 |
|  | EC:1.5.99.9 | mtd | methylenetetrahydromethanopterin dehydrogenase | K00319 | contig21302 | 474 |
|  | EC:1.5.99.11 | mer | coenzyme F420-dependent N5,N10-methenyltetrahydromethanopterin reductase | K00320 | contig28693 | 2122 |
|  | EC:1.12.98.1 | frhB | coenzyme F420 hydrogenase beta subunit | K00441 | contig11402 | 1948 |
|  |  |  |  |  | contig46651 | 759 |
|  | EC:2.3.1.101 | ftr | formylmethanofuran--tetrahydromethanopterin N-formyltransferase | K00672 | contig18430 | 2544 |
|  | EC:3.5.4.27 | mch | methenyltetrahydromethanopterin cyclohydrolase | K01499 | contig27684 | 1556 |
|  | EC:1.2.99.5 | fmdE | formylmethanofuran dehydrogenase subunit E | K11261 | contig17963 | 2870 |
|  |  |  |  |  | contig23327 | 748 |
|  |  |  |  |  | contig51241 | 301 |
| formate/acetate => methane | EC:2.1.1.86 | mtrA | tetrahydromethanopterin S-methyltransferase subunit A | K00577 | contig21544 | 740 |
|  |  | mtrD | tetrahydromethanopterin S-methyltransferase subunit D | K00580 | contig41237 | 839 |
|  |  | mtrE | tetrahydromethanopterin S-methyltransferase subunit E | K00581 | contig34552 | 862 |
|  |  | mtrH | tetrahydromethanopterin S-methyltransferase subunit H | K00584 | contig68116 | 427 |
|  |  |  |  |  | contig94477 | 321 |
| methanol/acetate => methane | EC:2.8.4.1 | mcrA | methyl-coenzyme M reductase alpha subunit | K00399 | contig65159 | 686 |
|  |  |  | methyl coenzyme M reductase system, component A2 | K00400 | contig51175 | 702 |
|  |  | mcrB | methyl-coenzyme M reductase beta subunit | K00401 | contig34913 | 615 |
|  |  | mcrG | methyl-coenzyme M reductase gamma subunit | K00402 | contig39855 | 263 |
|  | EC:1.8.98.1 | hdrA | heterodisulfide reductase subunit A | K03388 | contig20314 | 6141 |
|  |  |  |  |  | contig99644 | 1985 |
|  |  | hdrB | heterodisulfide reductase subunit B | K03389 | contig47344 | 539 |
|  |  |  |  |  | contig41258 | 526 |
| transporter/antiporter | EC:3.6.3.14 | ATPVA, ntpA | V-type H+-transporting ATPase subunit A | K02117 | contig28757 | 2817 |
|  |  |  |  |  | contig42488 | 2778 |
|  |  |  |  |  | contig44491 | 2254 |
|  |  |  |  |  | contig24907 | 2221 |
|  |  | ATPVB, ntpB | V-type H+-transporting ATPase subunit B | K02118 | contig11969 | 5323 |
|  |  |  |  |  | contig11473 | 2562 |
|  |  |  |  |  | contig03123 | 1242 |
|  |  | ATPVC, ntpC | V-type H+-transporting ATPase subunit C | K02119 | contig33613 | 507 |
|  |  |  |  |  | contig70894 | 387 |
|  |  | ATPVD, ntpD | V-type H+-transporting ATPase subunit D | K02120 | contig27107 | 1454 |
|  |  |  |  |  | contig42648 | 748 |
|  |  | ATPVI, ntpI | V-type H+-transporting ATPase subunit I | K02123 | contig30663 | 6032 |
|  |  |  |  |  | contig13571 | 5163 |
|  |  |  |  |  | contig03122 | 4208 |
|  |  |  |  |  | contig31895 | 3663 |
|  |  |  |  |  | contig26883 | 2482 |
|  |  |  |  |  | contig21477 | 2421 |
|  |  | ATPVK, ntpK | V-type H+-transporting ATPase subunit K | K02124 | contig52426 | 1453 |
|  |  |  |  |  | contig12817 | 832 |
|  |  | nhaA | Na+:H+ antiporter, NhaA family | K03313 | contig27893 | 652 |
|  |  |  |  |  | contig59060 | 644 |
|  |  | nhaC | Na+:H+ antiporter, NhaC family | K03315 | contig13965 | 2206 |
|  |  |  |  |  | contig78715 | 681 |
|  | EC:1.1.1.95 | serA, PHGDH | D-3-phosphoglycerate dehydrogenase | K00058 | contig21306 | 5160 |
|  |  |  |  |  | contig35025 | 1624 |
|  |  |  |  |  | contig103454 | 1468 |
|  |  |  |  |  | contig31123 | 1059 |
|  | EC:1.1.1.284 1.1.1.1 | frmA, ADH5, adhC | S-(hydroxymethyl)glutathione dehydrogenase / alcohol dehydrogenase | K00121 | contig13458 | 1357 |
|  |  |  |  |  | contig37322 | 363 |
|  |  |  |  |  | contig91859 | 271 |
|  | EC:1.2.7.1 | porA | pyruvate ferredoxin oxidoreductase, alpha subunit | K00169 | contig17431 | 4491 |
|  |  |  |  |  | contig41037 | 1353 |
|  |  | porB | pyruvate ferredoxin oxidoreductase, beta subunit | K00170 | contig14823 | 3761 |
|  |  |  |  |  | contig28861 | 958 |
|  |  |  |  |  | contig45355 | 543 |
|  |  | porD | pyruvate ferredoxin oxidoreductase, delta subunit | K00171 | contig79464 | 712 |
|  |  |  |  |  | contig72553 | 356 |
|  |  | porG | pyruvate ferredoxin oxidoreductase, gamma subunit | K00172 | contig95570 | 469 |
|  | EC:1.2.99.2 | cooS | carbon-monoxide dehydrogenase catalytic subunit | K00198 | contig65097 | 399 |
|  |  |  |  |  | contig97121 | 268 |
|  |  |  |  |  | contig79835 | 244 |
|  | EC:1.5.1.20 | metF | methylenetetrahydrofolate reductase (NADPH) | K00297 | contig14299 | 7258 |
|  |  |  |  |  | contig46459 | 3167 |
|  |  |  |  |  | contig23124 | 2423 |
|  |  |  |  |  | contig29794 | 2117 |
|  | EC:2.6.1.52 | serC, PSAT1 | phosphoserine aminotransferase | K00831 | contig113736 | 2097 |
|  |  |  |  |  | contig112089 | 891 |
|  |  |  |  |  | contig76011 | 300 |
|  | EC:2.7.9.2 | pps, ppsA | pyruvate, water dikinase | K01007 | contig12138 | 606 |
|  |  |  |  |  | contig34037 | 562 |
|  |  |  |  |  | contig49277 | 431 |
|  |  |  |  |  | contig93012 | 292 |
|  |  |  |  |  | contig30480 | 258 |
|  |  |  |  |  | contig93249 | 249 |
|  | EC:3.1.2.12 | frmB, ESD, fghA | S-formylglutathione hydrolase | K01070 | contig66571 | 379 |
|  | EC:3.1.3.3 | serB, PSPH | phosphoserine phosphatase | K01079 | contig97141 | 261 |
|  |  |  |  |  | contig97500 | 253 |
|  | EC:5.4.2.1 | gpmA, PGAM | 2,3-bisphosphoglycerate-dependent phosphoglycerate mutase | K01834 | contig05339 | 6119 |
|  |  |  |  |  | contig14980 | 1609 |
|  | EC:1.2.99.2 | coxS | carbon-monoxide dehydrogenase small subunit | K03518 | contig01872 | 2027 |
|  |  |  |  |  | contig01871 | 1352 |
|  |  |  |  |  | contig49204 | 930 |
|  |  | cutM, coxM | carbon-monoxide dehydrogenase medium subunit | K03519 | contig09479 | 12632 |
|  |  | cutL, coxL | carbon-monoxide dehydrogenase large subunit | K03520 | contig12450 | 10114 |
|  |  |  |  |  | contig13067 | 6070 |
|  |  |  |  |  | contig12465 | 4271 |
|  | EC:1.11.1.6 | katE, CAT | catalase | K03781 | contig09144 | 7296 |
|  |  |  |  |  | contig19966 | 2398 |
|  |  |  |  |  | contig41939 | 1921 |
|  | EC:1.11.1.21 | katG | catalase-peroxidase | K03782 | contig21913 | 2064 |
|  | EC:2.7.8.28 | cofD | LPPG:FO 2-phospho-L-lactate transferase | K11212 | contig107460 | 533 |
|  | EC:2.5.1.77 | cofG | FO synthase subunit 1 | K11780 | contig41873 | 406 |
|  | EC:2.1.1.- | mttB | trimethylamine methyltransferase | K14083 | contig30247 | 1610 |
|  |  |  |  |  | contig32955 | 662 |
|  | EC:1.12.99.- | mvhD, vhuD, vhcD | F420-non-reducing hydrogenase iron-sulfur subunit D | K14127 | contig35569 | 761 |
|  |  |  |  |  | contig86218 | 752 |
|  |  | mvhG, vhuG, vhcG | F420-non-reducing hydrogenase subunit G | K14128 | contig101853 | 1677 |
|  | EC:5.4.2.1 | gpmI | 2,3-bisphosphoglycerate-independent phosphoglycerate mutase | K15633 | contig14822 | 3782 |
|  |  |  |  |  | contig17942 | 1790 |
|  |  | gpmB | probable phosphoglycerate mutase | K15634 | contig104728 | 4401 |
|  |  |  |  |  | contig46477 | 1055 |
|  |  |  |  |  | contig24341 | 763 |
|  |  |  |  |  | contig35598 | 380 |
|  |  | apgM | 2,3-bisphosphoglycerate-independent phosphoglycerate mutase | K15635 | contig112318 | 5210 |
|  |  |  |  |  | contig10061 | 3050 |
|  |  |  |  |  | contig24560 | 2189 |

**Additional file Table S4**

| contig name | Letter^a^(bp) | Length^b^ | E value | Identity^c^(%) | Annotation | EC number | KO number | KEGG Pathway |
| --- | --- | --- | --- | --- | --- | --- | --- | --- |
| contig06058 | 32917 | 1557 | 8.00E-13 | 80 | D-lysine 5,6-aminomutase alpha subunit | EC:5.4.3.3 | K01844 | Amino Acid Metabolism |
| contig13066 | 11600 | 1278 | 2.00E-11 | 85 | chorismate synthase | EC:4.2.3.5 | K01736 | Amino Acid Metabolism |
| contig04473 | 10930 | 1845 | 4.00E-12 | 84 | hypothetical protein | EC:4.2.1.49 | K01712 | Amino Acid Metabolism |
| contig19589 | 13615 | 1413 | 8.00E-17 | 83 | cysteinyl-tRNA synthetase | EC:6.1.1.16 | K01883 | Amino Acid Metabolism/Genetic Information Processing/ |
| contig06075 | 30382 | 945 | 3.00E-18 | 80 | fructose-1,6-bisphosphate aldolase, class II | EC:4.1.2.13 | K01624 | Carbohydrate Metabolism |
| contig11446 | 18245 | 2361 | 7.00E-18 | 88 | formate acetyltransferase | EC:2.3.1.54 | K00656 | Carbohydrate Metabolism |
| contig00788 | 22540 | 3852 | 4.00E-17 | 83 | alpha amylase domain protein | EC:2.4.99.16 | K16147 | Carbohydrate Metabolism |
| contig06349 | 19413 | 951 | 1.00E-19 | 82 | ribose-phosphate pyrophosphokinase | EC:2.7.6.1 | K00948 | Carbohydrate Metabolism/Nucleotide Metabolism |
| contig15658 | 14596 | 1677 | 5.00E-12 | 85 | sigma 54 interacting domain protein | EC:3.4.21.53 | K04076 | Cellular Processes |
| contig04242 | 11880 | 1479 | 3.00E-10 | 82 | oligoendopeptidase F | EC: 3.4.24.- |  | Cellular Processes |
| contig07495 | 11639 | 1191 | 2.00E-17 | 85 | arsenical-resistance protein |  | K03325 | Cellular Processes and Signaling |
| contig12771 | 10710 | 1452 | 6.00E-11 | 83 | cation transporter |  | K03498 | Cellular Processes and Signaling |
| contig08798 | 9864 | 1059 | 9.00E-10 | 83 | PhoH-like protein PhoH1 |  | K06217 | Cellular Processes and Signaling |
| contig110034 | 24822 | 3699 | 4.00E-11 | 82 | transcription-repair coupling factor | EC: 3.6.1.- | K03723 | Energy Metabolism |
| contig110503 | 24438 | 1515 | 2.00E-19 | 86 | F0F1 ATP synthase subunit alpha | EC:3.6.3.14 | K02111 | Energy Metabolism |
| contig17513 | 10585 | 1896 | 4.00E-15 | 86 | putative Fe hydrogenase, electron-transfer subunit | EC:1.6.5.3 | K00335 | Energy Metabolism |
| contig06016 | 67686 | 1311 | 4.00E-22 | 87 | sn-glycerol 3-phosphate transport system substrate-binding protein |  | K05813 | Environmental Information Processing |
| contig07123 | 28745 | 831 | 3.00E-09 | 83 | ABC-2 type transporter |  | K09686 | Environmental Information Processing |
| contig25964 | 19963 | 1884 | 1.00E-10 | 82 | ABC transporter ATP-binding protein |  | K13892 | Environmental Information Processing |
| contig09188 | 18138 | 1269 | 4.00E-13 | 81 | branched-chain amino acid transport system substrate-binding protein |  | K01999 | Environmental Information Processing |
| contig11960 | 14284 | 927 | 1.00E-12 | 83 | branched chain amino acid ABC transporter permease |  | K01997 | Environmental Information Processing |
| contig09086 | 24389 | 1056 | 6.00E-19 | 83 | twitching motility protein |  | K02669 | Environmental Information Processing/Cellular Processes |
| contig06821 | 20671 | 1986 | 8.00E-15 | 84 | methyl-accepting chemotaxis sensory transducer |  | K03406 | Environmental Information Processing/Cellular Processes |
| contig03432 | 10651 | 1401 | 1.00E-18 | 85 | flagellar protein export ATPase FliI | EC:3.6.3.14 | K02412 | Environmental Information Processing/Cellular Processes |
| contig24245 | 9765 | 825 | 3.00E-09 | 81 | flagellar biosynthetic protein FliP | EC:3.1.1.3 | K02419 | Environmental Information Processing/Cellular Processes |
| contig107637 | 57966 | 2514 | 3.00E-13 | 84 | DNA gyrase subunit A | EC:5.99.1.3 | K02469 | Genetic Information Processing |
| contig06006 | 50132 | 2463 | 8.00E-23 | 83 | ATP-dependent Clp protease, ATPase subunit |  | K03696 | Genetic Information Processing |
| contig108077 | 35842 | 927 | 6.00E-20 | 88 | glycyl-tRNA synthetase subunit alpha | EC:6.1.1.14 | K01878 | Genetic Information Processing |
| contig07314 | 29111 | 2829 | 2.00E-10 | 84 | excinuclease ABC subunit A |  | K03701 | Genetic Information Processing |
| contig08075 | 27428 | 3522 | 4.00E-11 | 82 | chromosome segregation protein SMC |  | K03529 | Genetic Information Processing |
| contig03465 | 18369 | 795 | 7.00E-12 | 80 | RNA polymerase sporulation sigma factor SigG |  | K03091 | Genetic Information Processing |
| contig15382 | 16652 | 2352 | 2.00E-14 | 86 | Mername-AA223 peptidase | EC:3.4.24.- | K03798 | Genetic Information Processing |
| contig07120 | 12635 | 855 | 3.00E-16 | 86 | AraC family transcription regulator |  | K13653 | Genetic Information Processing |
| contig03563 | 12608 | 2838 | 2.00E-11 | 85 | DNA gyrase subunit A | EC:5.99.1.3 | K02469 | Genetic Information Processing |
| contig02876 | 10950 | 2079 | 3.00E-28 | 83 | translation initiation factor IF-2 |  | K02519 | Genetic Information Processing |
| contig03078 | 10395 | 2742 | 6.00E-14 | 85 | DNA gyrase subunit A | EC:5.99.1.3 | K02469 | Genetic Information Processing |
| contig07443 | 10149 | 1164 | 2.00E-38 | 85 | RNA polymerase, sigma 70 subunit, RpoD subfamily |  | K03086 | Genetic Information Processing |
| contig16833 | 9720 | 2976 | 2.00E-16 | 87 | glycine-tRNA ligase | EC:6.1.1.14 | K01878 | Genetic Information Processing |
| contig17740 | 16758 | 4341 | 2.00E-11 | 85 | DNA polymerase III, alpha subunit | EC:2.7.7.7 | K03763 | Genetic Information Processing |
| contig08805 | 18266 | 2505 | 2.00E-09 | 83 | preprotein translocase subunit SecA |  | K03070 | Membrane Transport |
| contig08379 | 14736 | 1092 | 5.00E-09 | 83 | polyphosphate kinase 2 | EC:2.7.4.1 | K09903 | Nucleotide Metabolism |
| contig00597 | 13475 | 2226 | 2.00E-11 | 82 | phosphoribosylformylglycinamidine synthase II | EC:6.3.5.3 | K01952 | Nucleotide Metabolism |
| contig11966 | 13103 | 633 | 5.00E-12 | 81 | uracil phosphoribosyltransferase | EC:2.4.2.9 | K00761 | Nucleotide Metabolism |
| contig09144 | 12632 | 1614 | 1.00E-27 | 81 | DNA polymerase III subunits gamma and tau | EC:2.7.7.7 | K02343 | Nucleotide Metabolism |
| contig10399 | 12538 | 2244 | 1.00E-12 | 85 | phosphoribosylformylglycinamidine synthase II | EC:6.3.5.3 | K01952 | Nucleotide Metabolism |
| contig22623 | 10444 | 2154 | 2.00E-23 | 83 | p ppGpp synthetase I GTP pyrophosphokinase , SpoT/RelA | EC:2.7.6.5 | K00951 | Nucleotide Metabolism |
| contig16348 | 13013 | 2373 | 5.00E-21 | 89 | polyribonucleotide nucleotidyltransferase | EC:2.7.7.8 | K00962 | Nucleotide Metabolism\Genetic Information Processing |
| contig16743 | 11817 | 1956 | 4.00E-12 | 81 | glycine reductase complex protein C large subunit | EC:1.21.4.2 | K10670 | unclassified |

a. The length of contig.

b. Alignment length.

c Percentage of identity of the aligned region.


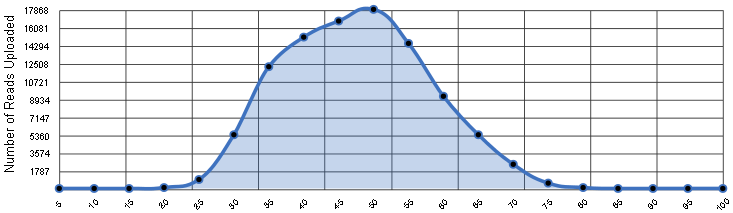


**Additional file Figure S1**


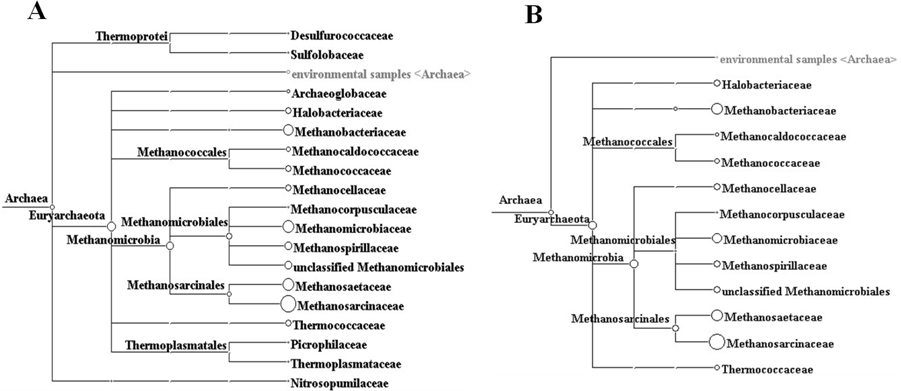


**Additional file Figure S2**

**
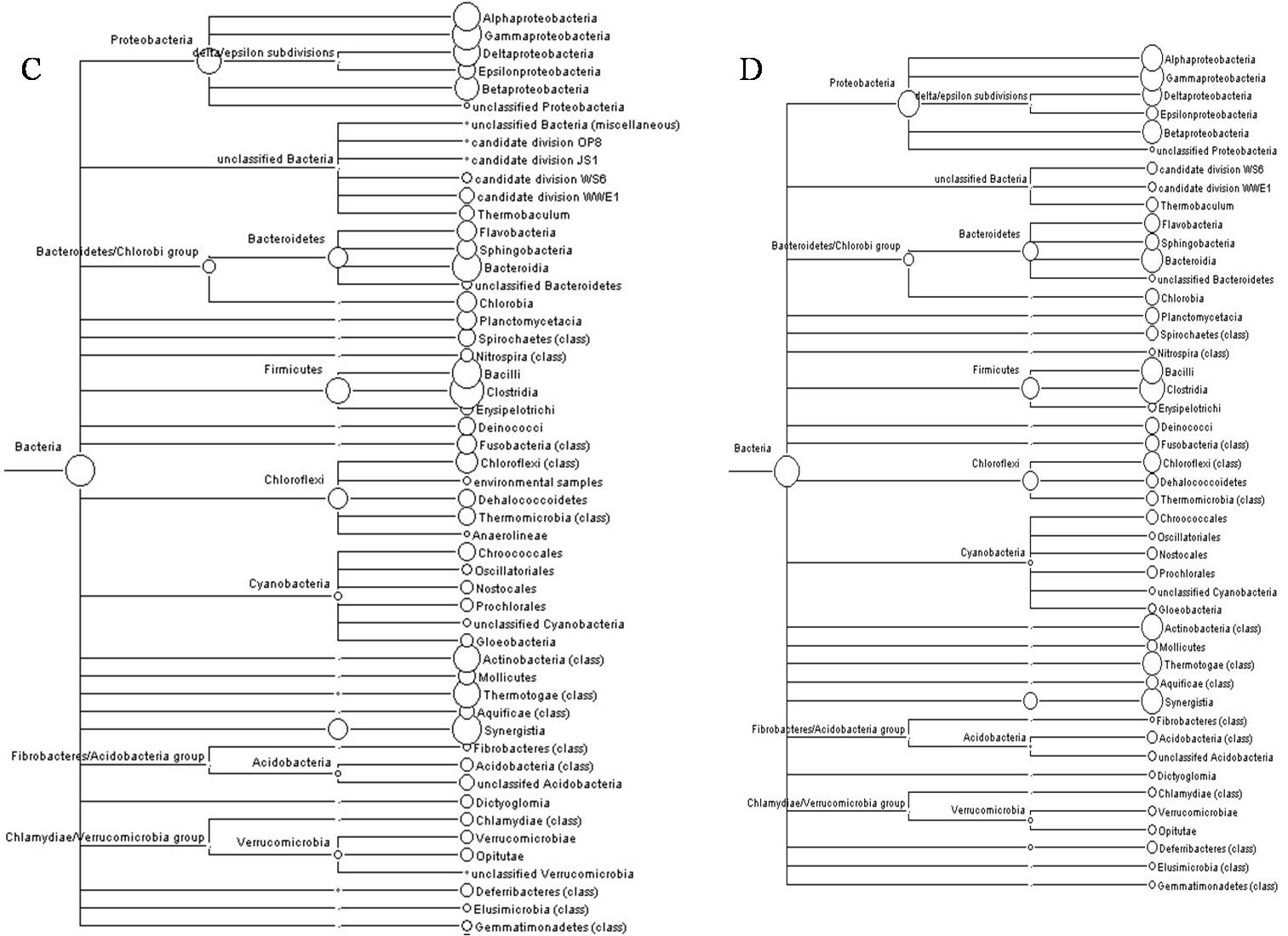
**

**Additional file Figure S2 (continued)**

**Additional file Figure S3**

**Additional file Figure S4**
